# Comparative separation methods and biological characteristics of human placental and umbilical cord mesenchymal stem cells in serum-free culture conditions

**DOI:** 10.1186/s13287-020-01690-y

**Published:** 2020-05-19

**Authors:** Xiao Yi, Feng Chen, Fenghua Liu, Qing Peng, Yang Li, Shao Li, Jiang Du, Yi Gao, Yifeng Wang

**Affiliations:** 1 Department of Gynecology, Zhujiang Hospital, Southern Medical University, Guangzhou, 510280 Guangdong Province China; 2 Department of Reproductive Medicine Center, Provincial Maternal and Child Health Hospital, Guangzhou, Guangdong Province China; 3 Department of Hepatobiliary Surgery II, Guangdong Provincial Research Center for Artificial Organ and Tissue Engineering, Guangzhou Clinical Research and Transformation Center for Artificial Liver, Institute of Regenerative Medicine, Zhujiang Hospital, Southern Medical University, Guangzhou, Guangdong Province China; 4 State Key Laboratory of Organ Failure Research, Southern Medical University, Guangzhou, China

**Keywords:** Mesenchymal stem cells (MSCs), Umbilical cord, Cell culture, Tissue explant method, Enzymatic digestion, Biological characteristics, Cell separation methods, Human placenta

## Abstract

**Background:**

Mesenchymal stem cells (MSCs) are considered to be an effective tool for regenerative medicine with promising applications for clinical therapy. However, incongruent data has been reported partially owing to their functional heterogeneity. To provide sufficient and suitable clinical seed cells derived from the placenta for MSC therapy, we compared the various current isolation methods, as well as the biological characteristics, of different human placenta mesenchymal stem cells (hPMSCs).

**Methods:**

We selected placentas from 35 informed donors and exploited three commonly used methods. MSCs were isolated from different parts of placental tissue including umbilical cord (UC), amniotic membrane (AM), chorionic membrane (CM), chorionic villi (CV), and deciduae (DC). The appropriate isolation methods for each type of hPMSCs were first assessed. The resulting five MSC types from the same individuals were identified based on their surface marker expression, proliferation capacity, transcriptome, differentiation, multipotency and karyotype.

**Results:**

All three methods successfully isolated the five hPMSC types from placental tissues. However, the UC-MSCs were most effectively separated via the tissue explant method, while the enzymatic digestion method was found to be more suitable for separating CV-MSCs, owing to its higher output efficiency compared to the other methods. Alternatively, the perfusion method was complicated and exhibited the lowest efficiency for cell isolation and uniformity. Furthermore, we determined that UC-MSCs and CV-MSCs express a higher level of paracrine cytokines and display much stronger proliferative capacity as well as superior extraction efficiency. Finally, karyotype analysis revealed that DC-MSCs are derived from the mother, while the other cell types are derived from the fetus. Moreover, the different hPMSCs exhibited unique gene expression profiles, which may prove advantageous in treatment of a broad range of diseases.

**Conclusions:**

hPMSCs from different sources are similar yet also unique. Our results describe the biological characteristics of five hPMSCs and provide insights to aide in the selection process of candidates for MSCs treatment. Overall, UC- and CV-MSCs appear to be ideal sources of primary MSCs for clinical treatment and future research.

## Background

Mesenchymal stem cells (MSCs) are ubiquitous throughout various tissues and possess self-renewal properties as well as multipotency and immunomodulatory functions. Due to these diverse properties, MSCs could serve as seed cells or gene therapy vectors, which offer promising application prospects in cell therapy and tissue engineering [1, 2]. Currently, MSCs derived from human bone marrow are the most thoroughly studied. However, the process of bone marrow extraction is invasive and can be accompanied by viral or bacterial contamination. Furthermore, owing to the wide range in donor age and individual variations, the proliferative and differentiation capacity of these cells is highly variable [3]. Although MSCs derived from fetal tissues show strong proliferative and differentiation capabilities, controversy over ethical issues limits its clinical application [4, 5]. It is, therefore, important to identify improved and sustainable MSC sources, while also determining the optima MSC yields for clinical application.

In recent years, researchers have attempted to isolate MSCs from fetal appendages including the placenta, umbilical cord (UC), cord blood, and amniotic fluid [6–9]. However, amniotic fluid-derived MSCs have been reported to exhibit high immunogenicity, while the extraction and separation of MSCs from the amniotic membrane (AM) is highly difficult. Alternatively, a much higher yield of MSCs with low immunogenicity was obtained from the UC and placenta compared to that of BMSCs and the other MSCs. Moreover, isolating MSCs from these sources minimizes any potential ethical restrictions.

The placenta is divided into four layers, namely the AM, chorionic membrane (CM), chorionic villi (CV), and deciduae (DC) [10–12]. Studies have shown that different placental tissue types possess unique proliferative and immunomodulatory properties as well as differences in paracrine cytokine secretion [13–19]. This suggests that MSCs derived from placental tissue types with distinguishable characteristics may achieve more efficacious therapeutic effects in specific diseases.

Herein, we analyzed the differences between MSCs derived from the UC and the different placental layers by using three common isolation methods, namely tissue block explant, enzymatic digestion, and umbilical vein perfusion methods. Also, as it is currently unclear whether these methods are equally effective in isolating MSCs from the different placental layers, we also assessed the individual feasibility and compared the output efficiency of the three methods. In addition, we described additional biological characteristics for each of the five MSC subsets. Finally, we identified the optimal large-scale culture method for UC and placental MSCs, while determining the most suitable disease range for their use to provide a basis for future research applications.

## Methods

We selected 35 placentas that met the requirements (maternal age less than 35 years, negative for infectious diseases, gestational age more than 35 weeks, regular birth examination, and no disease that interferes with placental vigor). Written informed consent was obtained from the donors. The study protocols were reviewed and approved by the Zhujiang Hospital review board and ethics committee of Zhujiang Hospital of Southern Medical University (approval number: 2019-KY-015-02). Thirty samples were treated with the tissue explant method and enzymatic digestion. Placental chorionic vascular MSCs were derived from five other samples after treatment with the umbilical vein perfusion method. The efficiency of different methods for isolation and culture of MSCs from the same placental parts was statistically analyzed, and the average passage time and yield (the number of primary MSCs that could be isolated from tissues of the same weight) were calculated. We selected MSCs from the same individuals (who provided placentas from which UC-MSCs were obtained by the tissue block culture method, and placental MSCs were obtained by enzymatic digestion) for biological characteristics analysis to avoid individual differences. Cells were cultured in serum-free media (Haoyang sc-82,013-G www.tbdscience.com) to minimize the individual differences associated with culture methods, which can impact results.

### Isolation and culture of hMSCs

#### Tissue explant method

The placenta was placed in a sterile box with 500 mL of physiological saline and transferred to a clean laboratory bench. Samples of UC, AM, CM, and CV of approximately 20 g were obtained. UC arteries were removed, and samples were cut into 1-cm strips and washed repeatedly with physiological saline. Each sample was homogenized in PBS into a fine granular shape (1–2 mm in diameter) using a hand-held electric homogenizer and rinsed repeatedly with PBS. Samples were centrifuged for 5 min at 150×*g*, the supernatant was removed, and the cells were washed twice with PBS with 1% streptomycin and placed as tissue pieces on the underside of a coated 75-cm^2^ flask with tissue sputum. The patch density was 1–2 pieces/cm^2^; a drop of medium was placed on each tissue block. Flasks were incubated at 37 °C in a 5% CO_2_, 95% humidity incubator for 4 to 6 h. Next, 2–4 mL of medium was slowly added along the wall of the flask, just over the UC tissue. The flasks were incubated for 7 days, after which two thirds of the volume was replaced with fresh medium. After 7 days, cells were observed once every 2 days. If the cells around a tissue block grew to a radius of 0.5 cm, it was marked under a microscope. A tip or pipette was used to gently dislodge the tissue block from the culture surface, which was floated in the culture flask. All tissue pieces were removed after the last collection time to collect migrated MSCs. When the cells reached 85–90% confluence, they were trypsinized for 30 s, neutralized with serum-free special neutralizing trypsin, centrifuged, and passaged at a 1:3 ratio, about 3000–4000 cells/cm^2^.

#### Enzymatic digestion method

Placental tissue was place in a dish and wash repeatedly with PBS. Approximately 20 g of tissue was treated as described for tissue explant samples. Next, 5 mL of tissue was placed in a 50 mL centrifuge tube to which 5 mL collagenase IV was added (collagenase IV with an activity of 255 U/mg can be formulated into a working solution at 2 mg/mL) and incubated at 37 °C, with agitation for approximately 60 min–90 min, until the tissue was digested to a sticky state. Next, 5 mL of trypsin (Gibco 25200056) was added, and the samples were shaken for 20–30 min. SFM medium was then added to a final volume of 50 mL and filtered through a 100-mesh sieve to filter out large pieces of tissue. Next, 20 mL of the filtrate was transferred to a new centrifuge tube with 40 mL of complete culture medium and centrifuged at 800×*g* for 20 min, after which 20 mL of supernatant was discarded while trying to avoid loss of the sticky supernatant. SFM medium was then added to a final volume of 40 mL and centrifuged at 800×*g* for 20 min, followed by removal of an additional 20 mL supernatant. SFM medium was again added to a final volume of 40 mL, after which the sample was transferred to a T75 flask and placed in a 5% CO_2_ incubator for 3–5 days. Once the cells were attached, half of the culture volume was replaced. Every 2 days thereafter, the cells were observed and passaged according to their growth. Generally, the cells were viable for 5–9 days with a large number of aggregates observed to form the germinal center. After 10–15 days, the cells in the culture flask grew to approximately 80–90% confluence and were passaged at 1:3, approximately 3000–4000 cells/cm^3^ of the flask.

#### Perfusion method

Complete placental tissue (required to maintain 25–35 cm of UC) was washed with PBS to fully remove blood on the surface of the placenta. An infusion suture from the umbilical vein was inserted and fixed with hemostatic forceps or suture. The other end of the infusion strip was connected to a 500-mL bottle of D-Hank’s medium pre-warmed at 37 °C and perfused with a peristaltic pump at a perfusion rate of 100–150 mL/min using a total of 3–5 bottles until the placental tissue became white. The tissue was then perfused with 400 mL of pre-incubated collagenase. The perfusion rate was 80–100 mL/min for approximately 20–30 min until the placental CM was granular. A constant temperature was used to maintain the perfusion enzyme temperature. Then, 400 mL of collagenase perfusate was collected. After neutralization, samples were centrifuged at 150×*g* for 5 min to collect the cells. Cells were washed once with PBS, and the concentration was adjusted to 1 × 10^8^ cells/L before transfer to a 75-cm^2^ dish with medium. The cells were statically cultured in an incubator at 37 °C with 5% CO_2_ and 95% humidity. When cell density reached 80–90%, cells were digested with 0.25% trypsin and subcultured at 1:3, approximately 3000–4000/cm^2^.

### Morphological observation of mesenchymal stem cells

After keeping the culture bottle static for 7 days, the morphology and growth of MSCs were observed under a microscope every day and photographed.

### Cell growth curves

MSCs at passage 3 were separated into single cell suspensions and plated at 1000 cells/well in 96-well plates in 100 μL of culture medium. There were seven groups with five wells in each group. One group was selected every 24 h. Using hemocytometer counting methods, cell numbers were averaged from five wells to construct a growth curve. Doubling time was estimated, and standardized data were used to compare subsequent cell viability.

### Cell viability by CCK-8 assay

MSCs were plated at 1000 cells/well in 96-well plates in 100 μL of culture medium. After the cells were attached, a 1/10 volume of medium from the CCK-8 Test Kit (Japan Tongren Chemical Research Institute) was added to each well in the dark and incubated for 2.5 h. Then, the liquid in the well was transferred to a new 96-well plate. Optical density (OD) was measured every 24 h with a microplate reader at 450 nm. For seven consecutive samples, the average OD of five wells was measured every day. The cell growth curve was constructed according to the OD of 10^4^ cells. Wells with culture medium only were used as a blank control group.

### Analysis of growth kinetics

The proliferation of MSCs from P3 to P9 was assessed (*n* = 3). MSCs from all sources were inoculated in a six-well culture plate at 1 × 10^4^ cells/well, and the cells were counted until they reached 100% confluency. Population doubling time (PDT) was calculated as follows: PDT = (CT × ln2)/ln (*N*_f_/*N*_i_), where CT is the cell culture time, *N*_i_ is the initial number of cells, and *N*_f_ is the final number of cells.

### Immunophenotypic identification

Third-generation MSCs that were close to confluency were trypsinized, washed, centrifuged, and diluted to 1 × 10^7^ cells/mL, and 200 μL of cell suspension was separately dispensed in seven flow tubes. The following were then added: FITC Mouse Anti-Human CD90 (5 μL), PE Mouse Anti-Human CD44 (5 μL), Percp-CyTM5.5 Mouse Anti-Human CD105 (5 μL), APC Mouse Anti-Human CD73 (5 μL), MSC positive isotype control cocktail (20 μL), and PE MSC negative isotype control cocktail (20 μL). All antibodies are components of the BD Stemflow hMSCs Analysis Kit (562245). Samples were incubated in the dark for 30 min and washed twice with 1 mL of PBS with centrifuging at 150×*g* for 5 min. Cells were resuspended in 500 μL PBS and tested by flow cytometry for surface markers.

### Adipogenic, osteogenic, and neurogenic differentiation potential of placental MSCs

MSCs at passage 3 were seeded in a 6-well plate at 3 × 10^4^ cells/L. When the cells reached 80–90% confluence, serum-free medium was removed, and pre-formed adipogenic (Stemcell Technologies), osteogenic (Stemcell Technologies), and neurogenic (Promocell GmbH) induction medium were separately added. Media in the adipogenic and osteogenic differentiation dishes were changed every 3 days for 10–15 days. Media in neurogenic differentiation dishes were changed every 48 h for 4–6 days. When lipid droplets appeared in the cytoplasm in the adipogenic induction group, the culture medium was carefully removed. Then, the samples were washed twice with PBS, fixed with 40 g/L paraformaldehyde for 20 min, and stained with oil red O. When the formation of a round calcified nodule was observed for the osteogenic induction group, cells were stained with alizarin red. On the first day of neurogenic differentiation, a significant morphological change was observed, and Nissl staining was performed at least 3 days after culture. MSC distribution in different placental tissues was observed by immunohistochemistry, and tissues of different levels of the placenta (UC, AM, CV, CM, and DC) were obtained. Conventional dehydration, sectioning, hematoxylin-eosin staining, and immunohistochemistry were performed, and images were obtained under a microscope.

### Submicroscopic structure of cells based on transmission electron microscopy (TEM)

The fresh cell pellet was immediately placed in 2.5% glutaraldehyde prepared in 0.1 M (pH 7.4) sodium bicarbonate buffer and fixed at 4 °C for 2 h. The pellet was washed three times with 0.1 M (pH 7.4) sodium bicarbonate buffer and placed in 1% citric acid prepared in the same buffer for 2 h (4 °C). The specimens were rinsed with double distilled water and dehydrated with 30%, 50%, and 70% ethanol for 10 min at 4 °C in sequence. Then, samples were soaked in 80%, 90%, and 95% acetone for 10 min each, and 100% acetone twice for 10 min. Samples were soaked in 1:1 acetone to resin for 2 h, followed by 1:2 acetone to resin for 2–4 h and 100% resin overnight. Each sample was mixed with embedding agent and incubated at 35 °C, 45 °C, and 60 °C for 24 h each to polymerize and harden the embedding agent. The specimens were sectioned at 60 nm with a cryostat and stained with uranium acetate dye for 10–20 min at room temperature. After washing with water, samples were stained with lead citrate dye for 15 min and washed twice with water. Samples were then dried and observed under a Hitachi H-7500 electron microscope.

### Karyotype analysis to determine the source of cells

UC-MSCs, AM-MSCs, CV-MSCs, CM-MSCs, and DC-MSCs were derived from three samples from the same placenta (male newborn). Each sample was assessed for logarithmic growth, condition, tightness, refractive index, and cell volume ≥ T25 or 60-mm plate. Two to 4 h before the termination of the culture, 20 μg/mL colchicine was added to bring the final concentration to 0.2 μg/mL, followed by incubation for 2 h. Cells were then collected and centrifuged at 150×*g* for 8 min. Eight milliliters of 0.075 mol/L KCl solution pre-warmed at 37 °C was added to the pellet, and each sample was incubated in a water bath at 37 °C for 25 min. Each sample was washed with 1 mL of freshly prepared fixative (methanol to glacial acetic acid = 3:1) and centrifuged at 150×*g* for 8 min. The supernatant was discarded, and 8 mL of fresh fixative was mixed with the cell pellet and incubated at room temperature for 20 min. The samples were centrifuged at 150×*g* for 8 min and washed twice with fixative solution. Two to three drops of each cell suspension were placed on a glass slide soaked in ice water and allowed to air dry. Cells were stained with Giemsa dyeing solution for 8 min, washed with water, and air-dried.

### Quantification of cell-secreted factors

Cells were seeded at 10,000 cells/cm^2^ in serum-free medium. After 72 h, cell-free supernatants were collected and stored at − 80 °C. Human ANG-1 (Abcam, UK, ab99972), hepatocyte growth factor (HGF) (Abcam, ab100534), IGF-I (Abcam, 100,545), transforming growth factor (TGF) (Abcam, ab100647), and vascular endothelial growth factor (VEGF) (Abcam, ab100662) were measured using an ELISA kit according to the manufacturer’s protocol.

### Transcriptome sequencing

The transcriptomic data were provided by Beijing Nuohe Zhiyuan Company (http://www.novogene.com). We performed statistical analysis of gene expression data to screen genes that were significantly differentially expressed in different states (SRA: SRP198666).

### Sample collection and preparation

#### RNA quantification and qualification

RNA degradation and contamination were monitored on 1% agarose gels. RNA purity was checked using the NanoPhotometer spectrophotometer (IMPLEN, USA). RNA concentration was measured using the Qubit RNA Assay Kit with a Qubit 2.0 Fluorometer (Life Technologies, USA). RNA integrity was assessed using the RNA Nano 6000 Assay Kit for the Bioanalyzer 2100 system (Agilent Technologies, USA).

#### Library preparation for transcriptome sequencing

A total of 3 μg RNA per sample was used as input material for RNA library preparation. Sequencing libraries were generated using the NEBNext Ultra RNA Library Prep Kit for Illumina (New England Biolabs, Inc., USA) following the manufacturer’s recommendations, and index codes were added to attribute sequences to each sample. Briefly, mRNA was purified from total RNA using poly-T oligo-attached magnetic beads. Fragmentation was carried out using divalent cations under elevated temperature in NEBNext first strand synthesis reaction buffer. First-strand cDNA was synthesized using random hexamer primers and M-MuLV reverse transcriptase (RNase H). Second-strand cDNA synthesis was subsequently performed using DNA polymerase I and RNase H. Remaining overhangs were converted into blunt ends via exonuclease/polymerase activities. After adenylation of the 3′ ends of the DNA fragments, NEBNext adaptors with hairpin loop structures were ligated to prepare for hybridization. To preferentially select cDNA fragments 250–300 bp in length, the library fragments were purified with AMPure XP beads (Beckman Coulter, USA). Then, 3 μL of USER Enzyme (New England Biolabs, Inc.) was used with size-selected, adaptor-ligated cDNA at 37 °C for 15 min, followed by incubation for 5 min at 95 °C. Then, PCR was performed with Phusion High-Fidelity DNA polymerase, universal PCR primers, and index primer. Finally, PCR products were purified with AMPure XP beads, and library quality was assessed on an Agilent Bioanalyzer 2100.

#### Clustering and sequencing (Novogene Experimental Department: http://www.novogene.com)

The clustering of the index-coded samples was performed on a cBot Cluster Generation System using TruSeq PE Cluster Kit v3-cBot-HS (Illumina) according to the manufacturer’s instructions. After cluster generation, the library preparations were sequenced on an Illumina HiSeq platform, and 125-bp/150-bp paired-end reads were generated.

### Data analysis

#### Quality control

Raw data (raw reads) in fastq format were first processed through in-house Perl scripts to remove reads containing adapters, reads containing poly-N, and low-quality reads. At the same time, Q20, Q30, and GC content were calculated. All downstream analyses used data that passed the initial quality control.

#### Read mapping to the reference genome

The reference genome and gene model annotation files were downloaded from the National Human Genome Research Institute website (https://www.genome.gov/). Paired-end cleaned reads were aligned to the reference genome using Hisat2 v2.0.5.

#### Quantification of gene expression level and differential expression analysis

featureCounts v1.5.0-p3 was used to count the reads mapped to each gene. Then, libraries were normalized by FPKM. Prior to differentially expressed genes (DEG) analysis, read counts were adjusted using the edgeR program package through one scaling normalized factor. Differential expression analysis of two conditions was performed using the edgeR package in R (3.18.1). The *p* values were adjusted using the Benjamini and Hochberg method. A corrected *p* value of 0.05 and absolute fold change of 2 were set as the threshold for significant differential expression.

#### Gene Ontology (GO) and KEGG enrichment analysis

For DEGs, GO enrichment analysis was implemented using the clusterProfiler (3.9) R package, in which gene length bias was corrected. GO terms with corrected *p* values less than 0.05 were considered significantly enriched. The KEGG is a database resource for understanding high-level functions and utilities of biological systems, such as the cell, the organism, and the ecosystem, from molecular-level information, especially large-scale molecular datasets generated by genome sequencing and other high-throughput experimental technologies (http://www.genome.jp/kegg/). We used the clusterProfiler R package to test the statistical enrichment of DEGs in KEGG pathways.

### Statistical analysis

Statistical analyses were performed using GraphPad Prism version 5.0 or SPSS22.0. Comparisons of parameters for more than three groups were performed by one-way analysis of variance (ANOVA) followed by Tukey’s test. Parametric data are expressed as the standard error of the mean (SEM). A value of *p* < 0.05 was considered statistically significant.

## Results

### Morphological observation and culture efficiency of MSCs

#### Tissue explant method

**Fig. 1:**
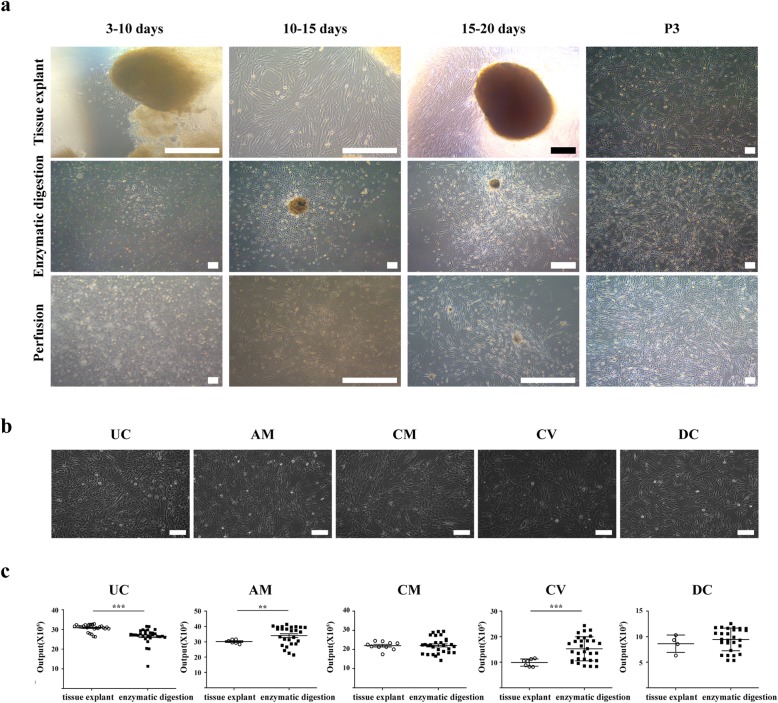
Morphological observation and yield of MSCs isolated from different tissues. **a** Morphological observation of MSCs based on isolation method (*n* = 35). Scale bar = 200 μm. **b**. Morphological observation of different types of MSCs isolated from different tissues (*n* = 30). Scale bar = 100 μm. **c** Yield of MSCs based on isolation method. UC-MSCs derived from tissue explant method had the highest yield, while the yield of AM-MSCs derived from enzymatic digestion was most abundant. All data were presented as mean ± SEM (*p < 0.05, **p < 0.01, ***p < 0.001)

Primary-cultured MSCs from the human UC and placenta were adherent. After culture for 3–10 days, cell adhesion occurred around some tissue blocks. For 10–15 days, the cells were centered on the tissue block and grew densely for 15–20 days, until cells covered the bottom of the bottle. The fibroblast-like cells were arranged in parallel or a spiral shape. After passage, they grew rapidly and could be passaged after 3 days (Fig. 1a, b). Morphology after 10 passages did not change significantly.

The success rate for UC and CM using the tissue explant method was 96.67% and 33.33%, respectively. The success rate for the AM was 20% because it is transparent, lighter, thinner, and easily disturbed after adherence. The CV has abundant blood vessels and red blood cells, which appeared to affect adherence success (23.33%). There are also many red blood cells in DC, and vitality was poor (13.33%). After primary isolation and culture from the UC, the passage time was the shortest, and the average number of cells per gram of tissue was the highest (Fig. 1c and Additional File 1: Table S1).

#### Enzyme digestion method

Using this method, primary-cultured human placental MSCs were adherent. After 72–96 h of culture, a small number of cells attached. A large number of cells adhered to form colonies with 1–2 locally dense germinal centers, after 10–12 days. The cells near the colony were fibroblast-like, long and spindle-shaped with cytoplasmic processes. The central cells of the colony were round, with abundant cytoplasm and large nuclei. The cells were spiral-shaped and multi-layered. There were a few epithelial cells, which proliferated after passage. The culture flask can be overgrown, and the cultured cells stably passaged after 3–4 days (Fig. 1a, b). There was no significant change in cell morphology before passage 10. The success rate from the five different tissues was more than 85%. The time to first passage for the CV was shortest, and the output from the AM was the most abundant (Fig. 1c and Additional File 1). Among tissue types, CV accounted for more than three quarters of the total placenta weight, with the most derived stem cells.

#### Perfusion method

After perfusion, some adherent cells were obtained. However, cell homogeneity was worse than with the enzymatic digestion method because there were few epithelial cells and flat stem cells. The operation was complicated and time-consuming, and the whole placenta was difficult to clean. Finally, the number of cells was much lower than with the enzyme digestion method (Fig. 1a). MSCs could be isolated from the five placental samples using the perfusion method. We analyzed 3–6 different human MSCs for biological characteristics, with UC-MSCs obtained by the tissue block culture method and placental MSCs obtained by enzymatic digestion.

### Growth kinetics of human placental MSCs

**Fig. 2:**
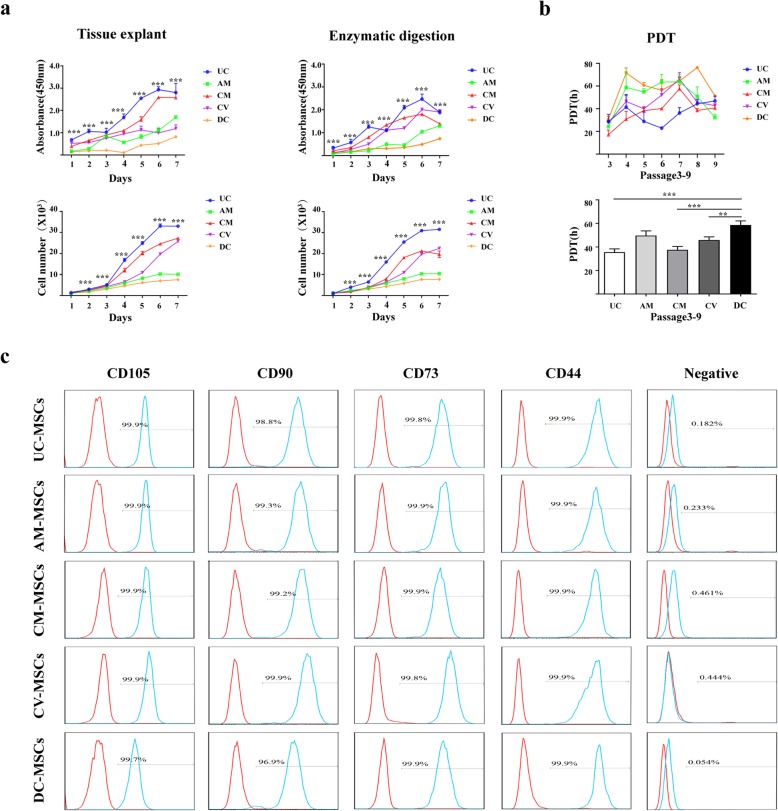
The proliferative potential and characterization of different types of MSCs derived from different tissues. **a** Growth kinetics of MSCs based on isolation method (*n* = 10). The data represent mean ± SEM (*p < 0.05, **p < 0.01, ***p < 0.001). **b** Comparison of population doubling time (PDT) of MSCs from different sources at P3–P9 by generation time and based on the average doubling time (*n* = 10 donors). The data as expressed as mean ± SEM (*p < 0.05, **p < 0.01, ***p < 0.001 (*n* = 3). **c** Flow cytometric analysis of the expression of surface markers on UC-MSCs and placental MSCs. The negative control cocktail includes CD34, CD19, CD11b, CD45, and HLA-DR

The growth kinetics of human placental MSCs were measured, showing that the primary-cultured cells entered an incubation period 1–2 days after inoculation and gradually began to adhere without evident amplification. After 3 days, the cells entered the logarithmic growth phase. In this phase, cell protrusions extended to the periphery. MSCs with two nuclear cell divisions were common, cell density increased, and many cells were connected. From 6 to 7 days, the cells gradually entered the plateau phase, and cell expansion decreased. The proliferative potential of MSCs occurred in the following order: UC > CM > CV > AM > DC (Fig. 2a and Additional File 1: Table S1). Accordingly, the proliferation rates of P3–9 generation MSCs (*n* = 3) and the average PDT were as follows: UC (35.60 ± 2.779 h), AM (49.67 ± 3.960 h), CM (37.62 ± 2.793 h), CV (45.83 ± 2.713 h), and DC (58.71 ± 3.408 h) (Fig. 2b). Thus, MSCs from different sources had different proliferation rates.

### Flow cytometric analysis

Flow cytometry showed that all cells highly expressed CD73, CD90, CD44, and CD105, with low expression of CD34, CD19, CD11b, CD45, and HLA-DR (Fig. 2c). Positive marker expression was more than 95%, whereas that of negative markers was less than 2%, which met the minimum standard of the International Society for Cell Therapy (ISCT) (Additional File 2: Table S2).

### Induction of differentiation among MSCs

**Fig. 3:**
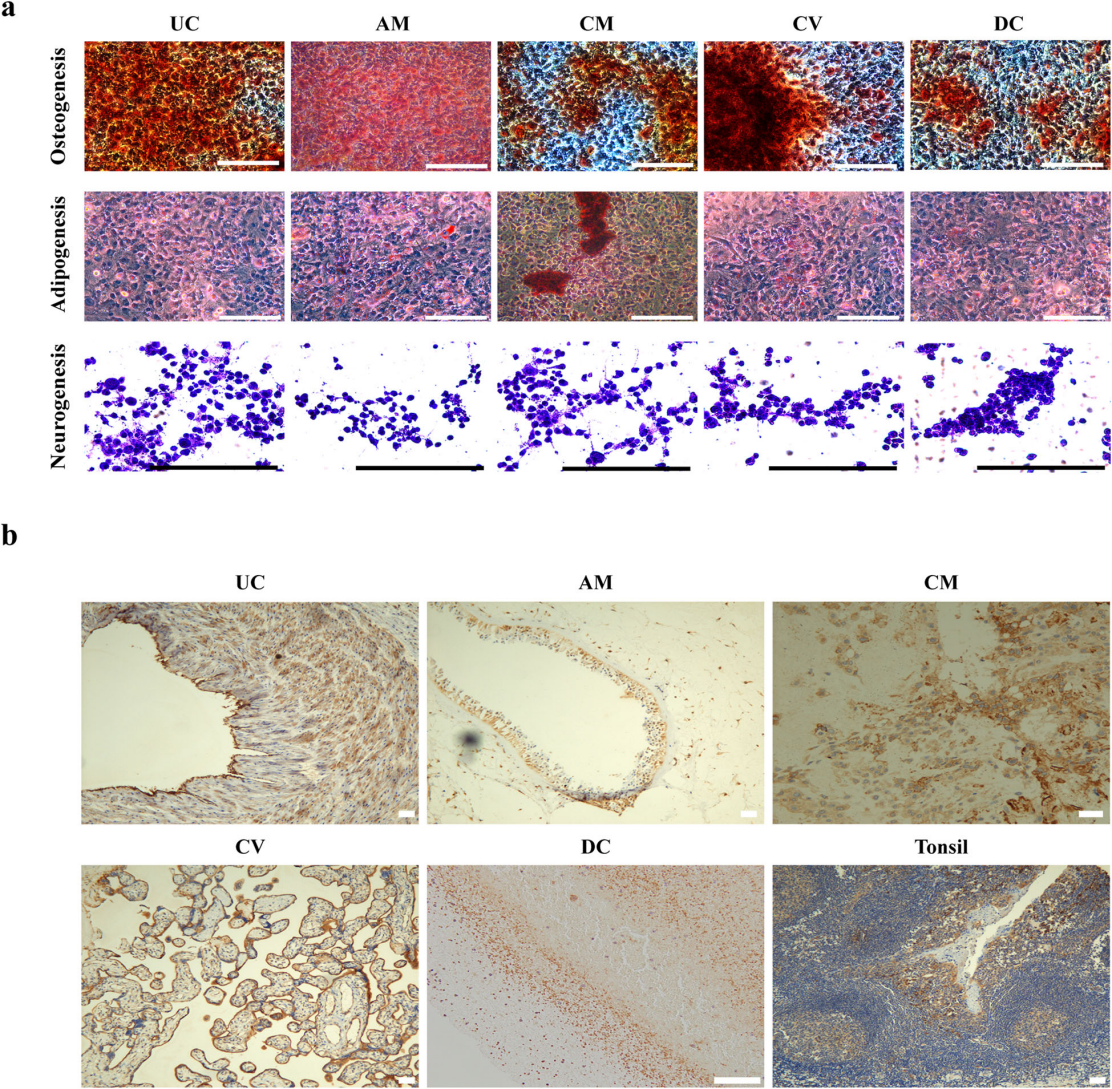
The differentiation potentials and immunohistochemical analysis of UC-MSCs and placental MSCs. **a** Phase-contrast microscopy (CK-2, OLYMPUS) was used to observe the morphology and differentiation of UC-MSCs and placental MSCs retrieved from different layers of the placenta: MSCs were positive for alizarin red (osteogenic differentiation), oil red O (adipogenic differentiation), and cresyl violet (neurogenic differentiation). Scale bar = 200 μm. **b** Immunohistochemical analysis (CD105) of different types of MSCs derived from different tissues (*n* = 3). Scale bar = 100 μm

The osteogenic induction of MSCs changed cells from a long fusiform to a cuboidal, cobblestone morphology. Cells stained with alizarin red exhibited colony growth and calcium nodules. Dense opaque masses formed between cells, and a large area of red staining appeared. The stained areas were large, and the cells showed clear osteogenic activity (Fig. 3a). Cell morphology after adipogenic induction began to change by 7 days from slender, spindle-shaped to hypertrophic cells. After 7–8 days, a small number of cytoplasmic lipid droplets formed, and a large number of fat cells were formed at 11 days. Oil red O staining confirmed lipid droplet formation (Fig. 3a). After 1 day of nerve cell induction, cell morphology became rounded and granulated. The cells were light blue after Nissl staining, and blue-black Nissl bodies were visible in the cells. There was no significant difference in differentiation or staining results between cells obtained by two different culture methods (Fig. 3a).

### Immunohistochemistry results

Antigen expression in different cell groups was determined by staining with a labeled CD105 antibody, and MSC localization in different placental tissues was assessed (tonsil was used a positive control). UC, AM, CM, and CV are rich in MSCs, and CD105 was expressed at the membrane (Fig. 3b). The cells isolated from UC, Wharton’s jelly, and CM were homogeneous. The AM is a transparent smooth membrane with blood vessels, so cells did not easily adhere, making AM-derived MSCs easily drift with the medium. The placental CV is formed by the extra embryonic mesoderm, and this layer is a solid tissue rich in blood vessels and mesenchymal components. The structure is complex but loose, and enzymes easily digested the MSCs in the tissue block. Therefore, the enzymatic digestion method had a high success rate and could separate a large number of MSCs.

### Transmission electron microscopy (TEM)

**Fig. 4:**
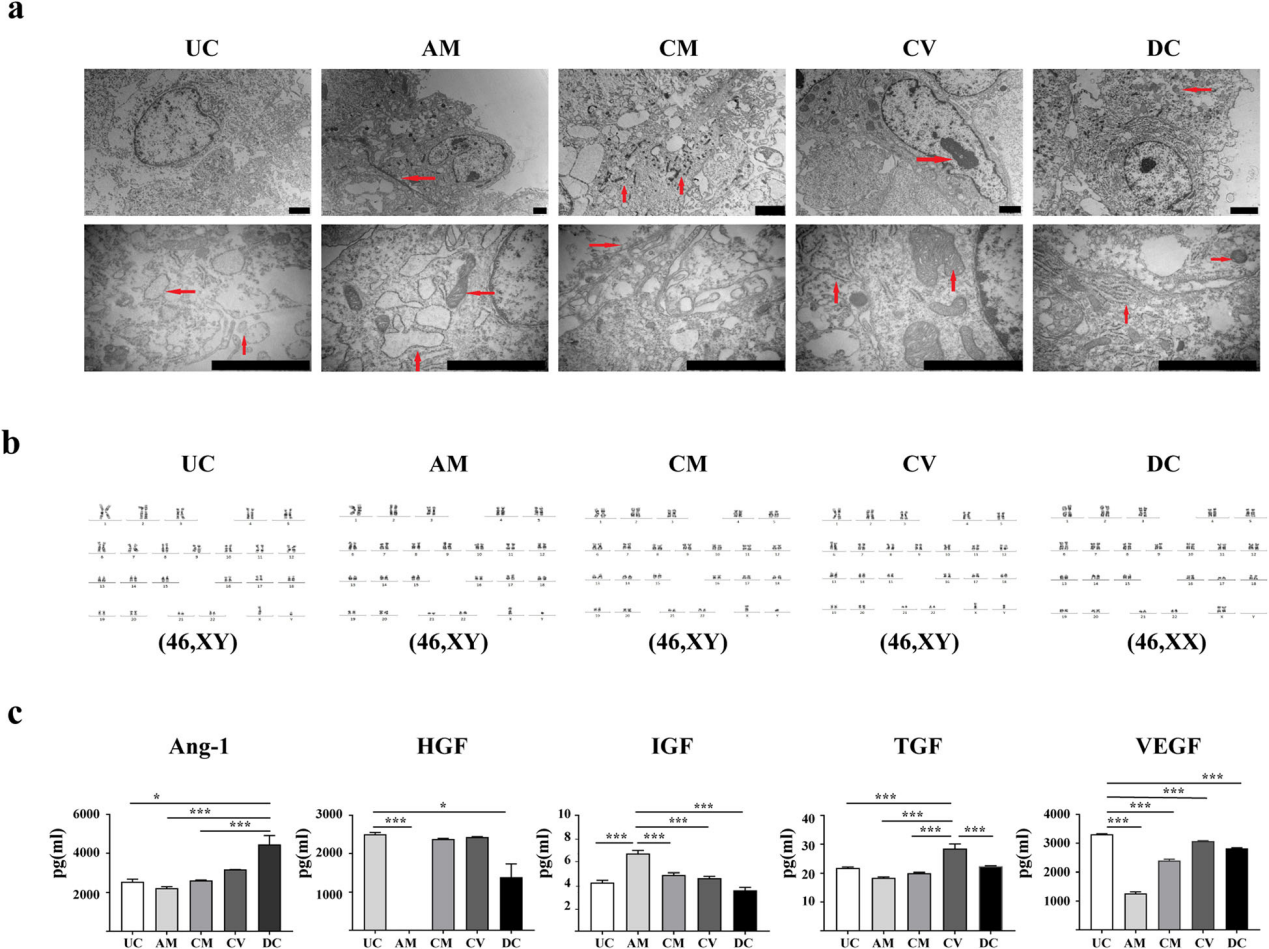
Comparison of the similarities and differences regarding different MSCs submicroscopic structure and karyotype and factors secreted from MSCs of different sources. **a** TEM images of MSCs derived from different tissues. The red arrows represent the abundant organelles and many membrane-secreted granules, which were spherical or pear-shaped, contained in the cytoplasm. Scale bar = 2 μm. **b** Karyotype analysis of MSCs from different origins. UC-MSCs, AM-MSCs, CM-MSCs, and CV-MSCs all exhibited a normal 46, XY karyotype, while DC-MSCs were maternal cells and exhibited a normal 46, XX karyotype (*n* = 10 per condition). **c** Comparison of the similarities and differences regarding different MSC factors secreted from MSCs of different sources. Error bars represent SEM (*n* = 10 per condition). (*p < 0.05, **p < 0.01, ***p < 0.001)

Based on TEM results (Fig. 4a and Additional File 3: Figure S1), most cells were long and fusiform, but some large, flat, irregular cells were also visible. Microvilli structures could be seen on the surface. The cells were mainly connected by tight and gap junctions, indicating adhesion and interactions between cells. Most cells were mononuclear, but were occasionally binuclear, with a large proportion of nucleoplasm and a clear nuclear membrane. The cells included inner and outer nuclear membranes with attached ribosomes on the outer surface of the outer membrane. The nucleolus was clear, and a small amount of heterochromatin was distributed in the periphery of the nucleus. Some cells exhibited nucleolar edge collection, where various RNAs are synthesized and rapidly released into the cytoplasm, indicating that the cells were metabolically active. There were many vacuoles in the cells, and the rough endoplasmic reticulum was visible in the cytoplasm. There were also many free ribosomes and mitochondria in the cells, and the mitochondria were clear. Autophagy was found to occur in some cells, and the chromatin was loose.

### Karyotype analysis

In five cases of the placenta with a male fetus, UC-MSCs, AM-MSCs, CM-MSCs, and CV-MSCs exhibited a normal 46, XY karyotype, whereas DC-MSCs were maternal cells and exhibited a normal 46, XX karyotype (Fig. 4b).

### ELISA results

There can be large differences in growth factors and cytokines secreted by MSCs from different sources. These paracrine factors include human angiopoietin-1 (Ang-1), HGF, insulin-like growth factor I (IGF-I), TGF, VCAM-1, and VEGF. Among the five tissues, the secretion of IGF-I was the highest in AM-MSCs, but these cells exhibited the lowest secretion of HGF, TGF, and VEGF. The secretion of the five factors from CM-MSCs was very high. The secretion of TGF in CV-MSCs was the highest. The secretion of the other four factors was also high, and the secretion of Ang-1 from DC-MSCs was the highest, whereas the secretion of IGF-I was the lowest in these cells. In UC-MSCs, the secretion of HGF and VEGF was the highest, whereas the secretion of Ang-1 was the lowest. CV- and UC-MSCs secreted higher levels of the different selected paracrine factors compared to AM-, CM-, and DC-MSCs. Differences between groups were statistically significant (Fig. 4c).

### Transcriptome sequencing analysis

**Fig. 5:**
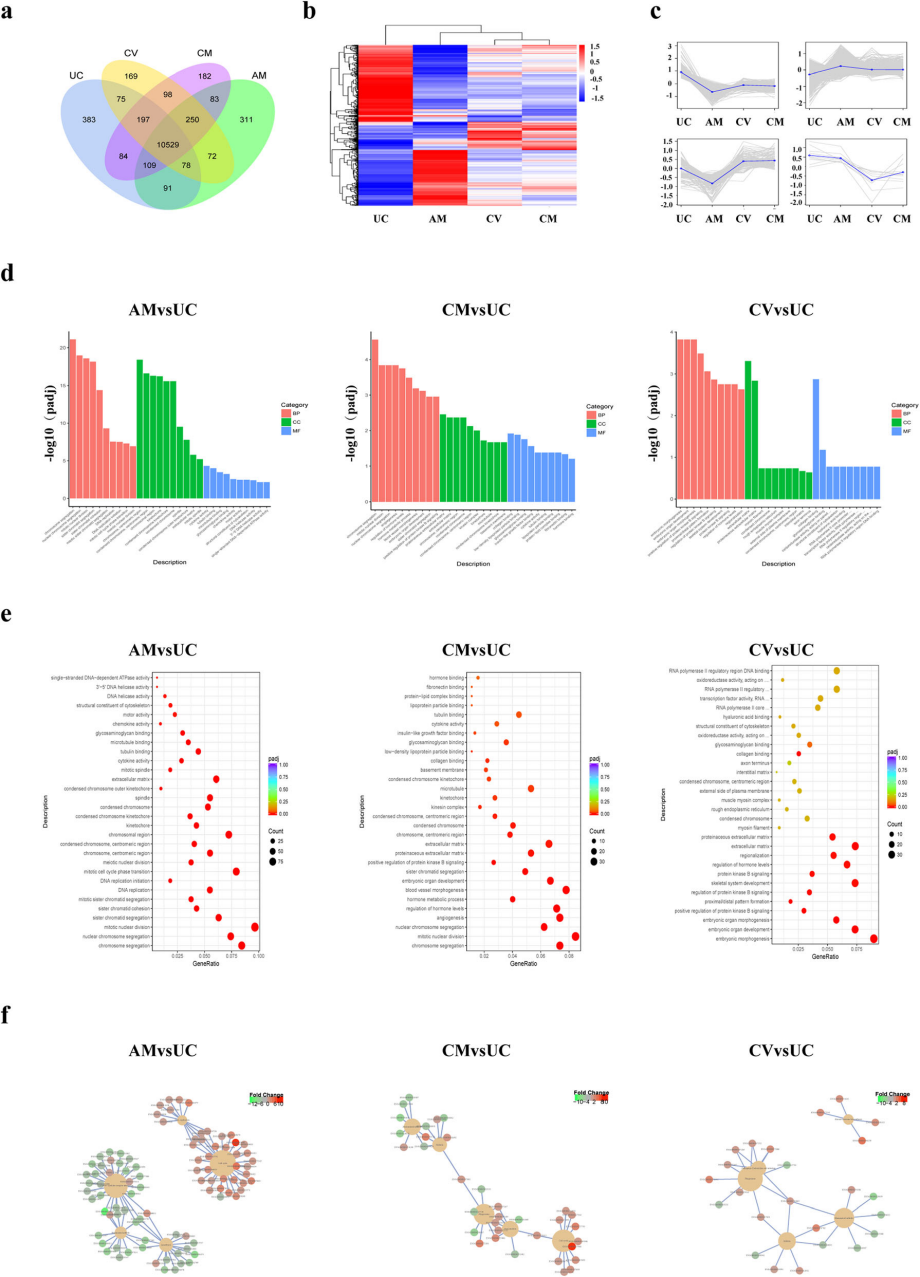
Transcriptome sequencing analysis of UC-MSCs, AM-MSCs, CM-MSCs, and CV-MSCs. **a** Venn diagram of differential gene expression among mesenchymal stem cells isolated from different sources. **b** Differential gene clustering analysis based on differentially expressed genes (DEGs) among MSCs isolated from different sources. **c** Line graph of expression pattern clustering among different experimental groups of MSCs from different sources. **d** Distribution of significantly different genes by GO term. GO analysis (**e**) and KEGG analyses (**f**) were utilized to evaluate the functions of these four types of MSCs based on their differentially expressed genes

We selected three fetal-derived human placental MSC and UC-MSC (control group) populations from the same mother for transcriptome sequencing analysis. The statistics and the number of DEGs for each combination and the criteria for screening differences are shown in Fig. 5a. (SRA: SRP198666).

#### DEG Venn diagram

There were 10,529 common genes (called hpcCS consensus genes) among the four different fetal-derived MSCs, which were UC–placental-consistent DEGs. The UC-MSC-specific region (UC-specific genes) had the most DEGs (383), the AM-MSC-specific region contained 311 DEGs, the CM-MSC-specific region had 182 DEGs, and the CV-MSC-specific region had 169 DEGs (Fig. 5a). These results suggested that hMSCs from the UC and placenta have similar gene expression patterns, but also specific genes.

#### Differential gene hierarchical clustering analysis

We used unsupervised hierarchical clustering to group gene expression normalized across rows. Note that heatmap colors can only be compared horizontally (the expression of the same gene in different samples), but not vertically (the expression of different genes in the same sample) (Fig. 5b). We used H-clustering to divide differential gene sets into several groups, and the genes in the same cluster had similar expression patterns under different processing conditions. Expression pattern clustering line diagrams based on different experimental groups are shown in Fig. 5c.

#### GO and KEGG pathway enrichment analysis

We next analyzed DEGs by GO and enrichment in KEGG pathways. We found that mRNA expression among the human placental MSCs from the four different sources differed significantly in terms of mitosis, regulation of hormone level hormone metabolism, angiogenesis of embryonic organs, binding of cytokine activity to tubulin, and activity of chemokine activity (Fig. 5d). From the results of GO enrichment analysis, the most significant terms were selected to generate scatter plots, as shown in Fig. 5e. We next plotted the five KEGG pathways with the most significant enrichment results, as shown in Fig. 5f.

## Discussion

The placental tissue is an ideal stem cell source and plays an outstanding role in MSCs application due to its wide range and convenience also with lower ethical restrictions. What is more, the collection process does not cause any damage to the mother or the newborn. There are various methods for the isolation of MSCs such like tissue explant, enzymatic digestion, density gradient centrifugation, flow cytometry, or others [18, 20–26]. The process of density gradient centrifugation performed so complicated which might has influence on the cell viability of MSCs. Flow cytometry can obtain high purity MSCs although seriously affects viability. Compared with the others, the tissue explant method operated easier with high available adherent cells besides the primary cells are mixed. Apart from its hard optimize time, the enzyme digestion method seems like to achieve a large-scale number of MSCs cells. However, enzyme concentration and digestion time are difficult to optimize. According to the structural differences between placenta and UC, we performed different methods to isolate and culture MSCs. In this study, we used a hand-held electric homogenizer to treat the UC and placental tissue, saving time, effort, and cost. Further, processing the tissue with a hand-held homogenizer avoids the need for cutting the tissue into tiny pieces, which limits specimen waste and dramatically improves separation efficiency.

The cells from human UC Wharton’s jelly are relatively simple, and after extensive washing, most red blood cells, granulocytes, and lymphocytes can be removed. Therefore, we recommend the tissue explant method to extract UC-MSCs and chorionic smooth muscle cells, as it is simple, economical, and convenient. It can also maximize cell viability and reduce the risks of contamination. Despite passaging the cells for ten generations, there was no significant change in proliferation activity and morphology, indicating that this method is suitable for isolating MSCs. CM-MSCs are robust and can be obtained by two methods, but the success rate of enzymatic digestion was significantly higher than that of tissue culture, and thus, the enzyme digestion method is preferred. The placental CV is a solid tissue-derived from extracellular mesoderm, which is rich in vascular and mesenchymal components. It has a complex structure, and the success rate of the tissue block explant method was low. The villus is loosely structured, and the surface is rich in MSCs, which are easily digested by enzymes. It is an ideal source for the large-scale clinical culture of MSCs. The technical difficulty associated with cultivating CV-MSCs is that the tissue is rich in blood vessels, which could affect cell attachment [24].

However, the use of red blood cell lysate can damage MSCs, and the reproducibility of lymphocyte separations is poor. Our results showed that the trophoblasts were washed and crushed and that the washing of finely divided tissue blocks can significantly reduce red blood cells and improve the success rate of enzyme digestion. The AM is a transparent and smooth film, with no blood vessels, but this tissue does not readily adhere to the vessel wall. AM-MSCs are mostly located on the surface of the amnion. They were also easy to digest into a single cell suspension, and thus, the enzymatic digestion method was found to have a higher success rate. DC-MSCs have reduced viability compared to UC-MSCs, and have low migration ability, and thus do not easily migrate from the tissue block. However, the decidual tissue structure is loose and can be easily digested by enzymes. Therefore, enzymatic digestion is more suitable. The UC and CV are therefore ideal sources for clinical large-scale MSC culture, as they are rich in stem cells and associated with the highest extraction efficiency.

Based on this, we then analyzed the characteristics of the five different MSCs obtained from UC and placenta. We found that UC-, AM-, CM-, and CV-MSCs all expressed similar immune phenotypes and could differentiate into adipocytes, osteoblasts, and neuroblasts, meeting the minimum standards set by the ISCT in 2006. Moreover, it was previously reported that the order of growth rate for the cells is (fastest to slowest) UC-, AM-, CP-, and DP-MSCs [27], which agrees with our findings. However, another study reported that although similar numbers of viable cells were observed in AM-, CP-, and UC-MSC cultures within the first 3 days, significant differences began to emerge beginning on the fourth day, with CP-MSCs exhibiting higher proliferative capacity than that of the other two groups [28]. Further, the use of different cell generations, methods for cell isolation, and culture conditions, as well as individual differences in placenta origin, may impact the experimental results. Nevertheless, the general trend is for UC- and chorionic-derived MSCs to exhibit stronger proliferative capacity compared to other MSC subtypes.

We also performed karyotype analysis for the five types of placental tissue-derived MSCs and found that UC-MSCs, AM-MSCs, CM-MSCs, and CV-MSCs were obtained from the fetus whereas DC-MSCs were derived from the maternal tissue. In addition, we observed more mitochondrial in the placenta-derived MSCs compared to UC-MSCs by using the electron microscopy. Further, MSCs from placenta had more abundant ribosomes, and cytokine secretion ability was stronger than those from the UC. Moreover, placenta-derived MSCs had more abundant ribosomes and paracrine cytokines than those from the UC.

Paracrine may be an important mechanism for MSCs in the application of curing diseases. Studies have shown that AM-MSC have effective treatment on premature ovarian failure partly owing to the high secretion levels of PGE2 and TGF-β1 [29]. CV-MSC has a potential angiogenic effect which may be related to its HGF and VCAM-1 [30] secretion. Studies also demonstrated that adult-derived DC-MSCs could be used for severe lower limb defects due to their high secretion of VEGF and Ang-1 [31]. In our study, we found that paracrine cytokines secreted by UC-MSCs and placenta-derived MSCs were distinct. Similarly, ANELISE BERGMANN ARAÚJO reported that compared to AM-, CP- and DP-MSCs, UC-MSCs secreted higher levels of a wide range of select paracrine factors. Thus, UC-MSCs may be a source for cell therapy to treat other diseases [27]. This is similar to our findings as compared to AM-, CM-, and DP-MSCs, CV- and UC-MSCs secreted higher levels of multiple paracrine factors. We speculate that CV- and UC-MSCs may, therefore, be more suitable sources for cell therapy to treat other diseases. The CV tissue accounts for more than 90% of the total weight of the placenta. Hence, its superior cytokine secretion properties might be related to the material exchange and secretory function from the mother to the fetus during development.

The global gene expression pattern analysis among MSCs derived from different neonatal tissues has been reported previously [32, 33]; however, as far as we know, there has been no comparative analysis of gene expression patterns for the five sources of cells cultured in SFM. Considering that UC-MSCs exhibit different gene expression patterns when cultured in SFM [34], our data was the first to identify the differentially expressed gene patterns among these MSCs cultured under the serum-free GMP condition. Based on a comparison of transcriptome sequencing analysis, we identified common and specific genes for the human placenta and UC sources. Our results indicate that hMSCs from the UC and placenta have similar gene expression patterns, but also some differences. Such specific genes were involved in cell cycle, cell division, cell death, cell growth, and development. These genes also play a role in transcriptional regulation, DNA repair, DNA replication, and chromosomal stability, which are an important part of cell or subcellular component movement, cell communication, cell-tissue projections, cytokine secretion, and hormone metabolism. The results of transcriptome sequencing analysis explain, to some extent, the differences in biological characteristics among MSCs from different sources. We believe that these specific DEGs could provide clues to study biological markers and their corresponding functions (Additional File 4: Table S3).

The sources of human placental MSCs are stable and are gradually being used as seed cells for regenerative medicine. This study used serum-free medium to compare isolation and culture methods for MSCs from UC and different levels of the placenta, thus establishing a complete system for cell isolation, culture, and identification. Moreover, we analyzed biological characteristics among the five different sources of cells with respect to cell proliferation, cell viability, immunophenotypes, cell differentiation, submicroscopic structures, karyotype analysis, paracrine factors, and transcriptome sequencing. These tissues provide sufficient sources of MSCs for clinical applications, and to some extent, the results of this study could be used to select a suitable cell source for different needs. Better treatment effects may be obtained if both the characteristics of MSCs from different sources and the goal of the clinical application are considered. A limitation of this study is that we only studied the biological characteristics of different MSCs at the cellular level and did not compare UC- and placenta-derived MSCs from animal tests or clinical trials.

## Conclusions

Cells from different sources are similar, but also different. Overall, UC- and CV-MSCs are ideal sources of primary MSCs for clinical treatment and research. Our results identify the biological characteristics of the five hPMSCs and provide a certain idea for the selection of candidate for MSCs treatment.

## Supplementary information

**Additional file 1 : Table S1**. Success rate and mesenchymal stem cell extraction efficiency from different tissues.

**Additional file 2 : Table S2**. Expression of surface antibody of from passage 3 mesenchymal stem cells from different sources at passage 3.

**Additional file 3 : Figure S1**. TEM images of MSCs derived from different tissues.

**Additional file 4.** Total number of differentially expressed genes by transcriptome sequencing analysis.

## Data Availability

Research data are not shared.

## Abbreviations

AM: Amniotic membrane
CM: Chorionic membrane
CV: Chorionic villi
DC: Deciduae
DEGs: Differentially expressed genes
GO: Gene Ontology
ISCT: International Society for Cell Therapy
KEGG: Kyoto Encyclopedia of Genes and Genomes
MSCs: Mesenchymal stem cells
TEM: Transmission electron microscopy
UC: Umbilical cord
