# Supplementary material for: Comparative separation methods and biological characteristics of human placental and umbilical cord mesenchymal stem cells in serum-free culture conditions
Source: Stem Cell Res Ther. 2020 May 19;11:183. doi: 10.1186/s13287-020-01690-y (PMC7238656; doi:10.1186/s13287-020-01690-y)
Supplement: Supplementary file 1 — Additional file 1 : Table S1. Success rate and mesenchymal stem cell extraction efficiency from different tissues. [file 13287_2020_1690_MOESM1_ESM.docx]

**Additional File 1.** Success rate and mesenchymal stem cell extraction efficiency from different tissues.

|  | | UC | AM | CM | CV | DC |
| --- | --- | --- | --- | --- | --- | --- |
| Tissue  explant  method  (n = 30) | Rate | 96.67% | 20.00% | 33.33% | 23.33% | 13.33% |
|  | Time (h) | 17.03 ± 0.52 | 21.67 ± 1.05 | 20.40 ± 0.65 | 21.57 ± 0.65 | 26.5 ± 1.19 |
|  | Output (×105) | 30.84 ± 0.34 | 30.17 ± 0.52 | 21.99 ±0.67 | 9.88 ±0.54 | 8.63 ±0.85 |
| Enzymatic  digestion  method  (n = 30) | Rate | 100% | 86.67% | 96.67% | 100% | 93.33% |
|  | Time (h) | 18.90 ± 0.57 | 22.54 ± 0.40 | 21.69 ± 0.54 | 16.83 ± 0.56 | 24.29 ± 0.62 |
|  | Output (×105) | 26.64 ± 0.71 | 34.03 ± 1.19 | 22.12 ± 0.77 | 15.26 ± 0.86 | 9.46 ± 0.42 |

“Time” means the first passage time. Error is displayed as SEM.
