# Supplementary material for: Comparative separation methods and biological characteristics of human placental and umbilical cord mesenchymal stem cells in serum-free culture conditions
Source: Stem Cell Res Ther. 2020 May 19;11:183. doi: 10.1186/s13287-020-01690-y (PMC7238656; doi:10.1186/s13287-020-01690-y)
Supplement: Supplementary file 2 — Additional file 2 : Table S2. Expression of surface antibody of from passage 3 mesenchymal stem cells from different sources at passage 3. [file 13287_2020_1690_MOESM2_ESM.docx]

**Additional File 2.** Expression of surface antibody obtained from mesenchymal stem cells from different sources at passage 3.

|  | | CD105 | CD73 | CD90 | CD44 | Negative |
| --- | --- | --- | --- | --- | --- | --- |
| Tissue  explant  method  (n = 6) | UC | 99.5 ± 0.5 | 99.3 ± 0.4 | 98.6 ± 1.1 | 99.4 ± 0.5 | 0.7 ± 0.3 |
|  | AM | 97.5 ± 1.1 | 96.7 ± 0.6 | 97.7 ± 1.5 | 97.8 ± 0.6 | 1.1 ± 0.3 |
|  | CM | 98.8 ± 0.9 | 98.8 ± 0.6 | 98.4 ± 0.8 | 99.1 ± 1.0 | 0.6 ± 0.4 |
|  | CV | 99.3 ± 0.7 | 98.6 ± 0.6 | 98.0 ± 0.8 | 98.8 ± 0.5 | 0.8 ± 0.3 |
|  | DC | 98.0 ± 0.7 | 98.5 ± 0.4 | 99.0 ± 0.8 | 98.5 ± 0.3 | 0.6 ± 0.3 |
| Enzymatic  digestion  method  (n = 6) | UC | 99.3 ± 0.6 | 99.3 ± 0.4 | 99.6 ± 0.4 | 98.5 ± 1.0 | 0.4 ± 0.1 |
|  | AM | 97.5 ± 1.1 | 96.7 ± 0.8 | 97.6 ± 1.4 | 98.0 ± 1.3 | 0.9 ± 0.2 |
|  | CM | 99.0 ± 0.5 | 99.0 ± 0.5 | 99.5 ± 0.4 | 99.2 ± 0.7 | 0.4 ± 0.1 |
|  | CV | 99.0 ± 0.6 | 98.7 ± 0.7 | 99.0 ± 0.8 | 99.2 ± 0.5 | 0.4 ± 0.2 |
|  | DC | 98.6 ± 0.5 | 98.8 ± 0.6 | 98.8 ± 0.6 | 97.8 ± 1.4 | 0.5 ± 0.2 |
| Perfusion method | | 97.3 ± 1.3 | 98.0 ± 1.0 | 98.0 ± 0.8 | 97.4 ± 1.8 | 0.8 ± 0.4 |

UC, umbilical cord; CM, chorionic membrane; CV, chorionic villi; AM, amniotic membrane; DC, decidua.
