# Supplementary material for: Comparative separation methods and biological characteristics of human placental and umbilical cord mesenchymal stem cells in serum-free culture conditions
Source: Stem Cell Res Ther. 2020 May 19;11:183. doi: 10.1186/s13287-020-01690-y (PMC7238656; doi:10.1186/s13287-020-01690-y)
Supplement: Supplementary file 3 — Additional file 3 : Figure S1. TEM images of MSCs derived from different tissues. [file 13287_2020_1690_MOESM3_ESM.pdf]

Figure S1 TEM images of MSCs derived from different tissues.

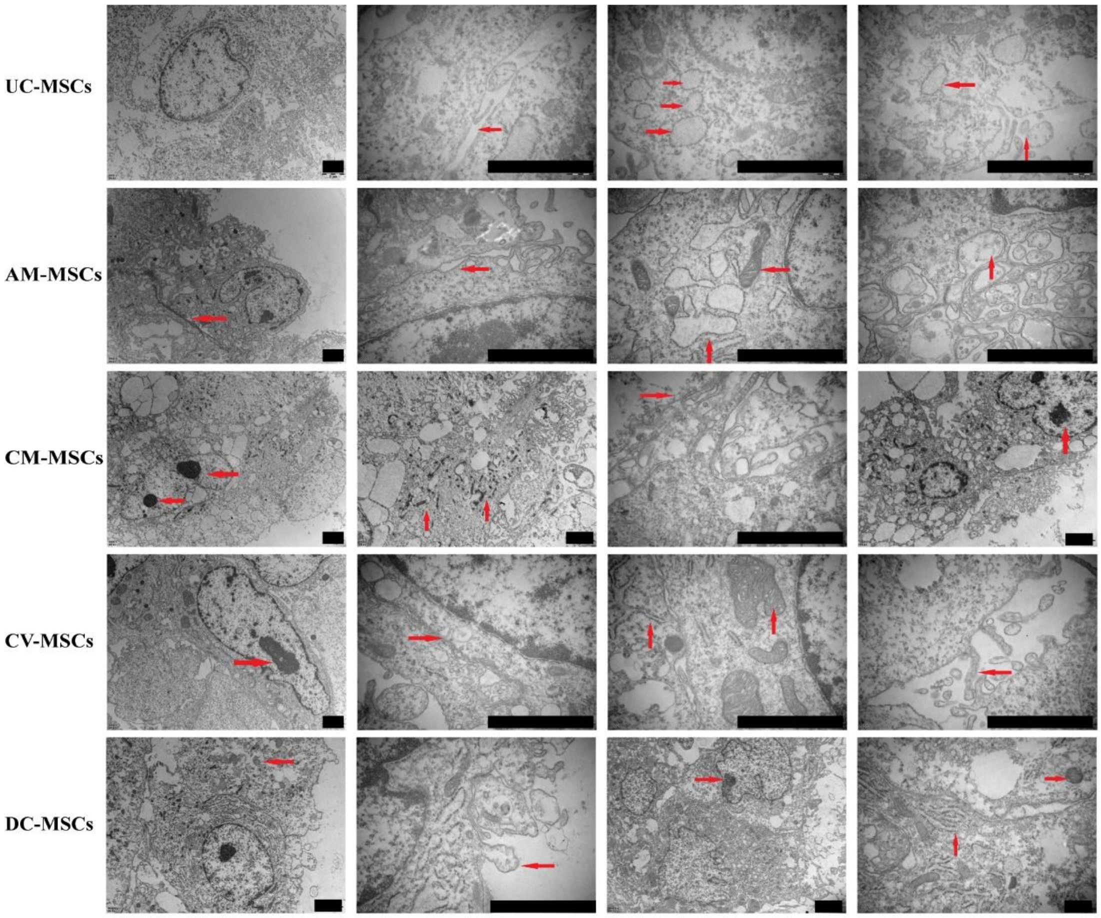

Image showing that the nucleolus was clear, and there were many vacuoles in the cell. Microvilli structures could be seen on the surface. The cells were mainly connected by tight and gap junctions. And there were many membrane-secreted granules and organelles in the cells, such as rough endoplasmic, tubular crista mitochondria, and Golgi apparatus. n=3.Scale bar=2μm.
