# Supplementary material for: Comparative separation methods and biological characteristics of human placental and umbilical cord mesenchymal stem cells in serum-free culture conditions
Source: Stem Cell Res Ther. 2020 May 19;11:183. doi: 10.1186/s13287-020-01690-y (PMC7238656; doi:10.1186/s13287-020-01690-y)
Supplement: Supplementary file 4 — Additional file 4. Total number of differentially expressed genes by transcriptome sequencing analysis. [file 13287_2020_1690_MOESM4_ESM.docx]

**Additional File 4.** Total number of differentially expressed genes by transcriptome sequencing analysis.

| Compare | All | Up | Down | Threshold |
| --- | --- | --- | --- | --- |
| AMvsUC | 1217 | 553 | 664 | edgeRpadj<0.05 \|log_2_FoldChange\|>1.0 |
| CMvsUC | 553 | 278 | 275 | edgeRpadj<0.05 \|log_2_FoldChange\|>1.0 |
| CVvsUC | 547 | 289 | 258 | edgeRpadj<0.05 \|log_2_FoldChange\|>1.0 |

UC, umbilical cord; CM, chorionic membrane; CV, chorionic villi; AM, amniotic membrane; DC, decidua.

“Compare” column describes the tissue types; Up, the number of upregulated differential genes; Down, the number of downregulated differential genes; Threshold, the criteria necessary for significance, software used for statistical testing, and fold-expression threshold
